# An in vitro confirmation of the ethonopharmacological use of *Senna* plants as anthelmintic against rumen fluke *Paramphistomum gracile*

**DOI:** 10.1186/s12917-019-2094-3

**Published:** 2019-10-22

**Authors:** Saptarshi Roy, Larisha Mawkhlieng Lyndem

**Affiliations:** Parasitology Research Laboratory, Department of Zoology, Visva-Bharati University, Santiniketan, West Bengal 731235 India

**Keywords:** *Senna*, Trematode, Inhibition, Tegument, Traditional medicine

## Abstract

**Background:**

Paramphistomosis is a pathogenic disease of domesticated ruminants, causing great economic loss in dairy industry and meat production. It is considered as a neglected tropical disease with highest prevalence throughout tropical and subtropical regions, particularly in Africa, Asia, Europe, and Australia. There are few trematocidal drugs available in the market. Most are resistant and have elevated side effects. Therefore, alternative trematocidal drugs need to discover. This study was conducted to evaluate three plants leaf extracts (from *Senna alata*, *S. alexandrina,* and *S. occidentalis*) as effective remedies against gastrointestinal trematode parasite (*Paramphistomum gracile*) of ruminants*.* Live adult parasites were collected in 0.1 M phosphate-buffered saline (PBS) from fresh autopsied goat’s rumen. Parasites were incubated in leaf extracts of *S. alata*, *S. alexandrina* and, *S. occidentalis* individually and in combination (1:1) ratio at 37 ± 1°C. Treatment media contain extracts at different concentrations (10, 20 and 40 mg/mL) in 10 mL of 0.1 M PBS with 1% dimethylsulphoxide (DMSO). Parasites in control group were incubated in PBS without extract. The efficacy of three *Senna* extracts was evaluated on the basis of dose-dependent motility and mortality of the trematode. Immediately after paralysis, all treated parasites were collected for histology, SEM and biochemical study.

**Results:**

Dose-dependent efficacy was observed in terms of motility and time of mortality in all treated parasites after exposure in various concentrations of the *Senna* plant extracts. *S. occidentalis* and *S. alexandrina* showed better efficacy in combination than comparing with individual treatment groups. Histological study and scanning electron microscopic observations revealed conspicuous deformity of surface architecture in all treated parasites. Scanning electron micrographs also revealed shrinkage, vacuolization, infoldings and blebbings on the body surface of treated worms. Activities of tegumental enzymes were inhibited in all treatment groups compared to control.

**Conclusion:**

The overall findings from this study revealed that all three *Senna* leaf extracts individually and in combination showed potential antitrematocidal activity against *Paramphistomum gracile* by damaging body tegument and neural propagation. Thus, this study confirmed that all three *Senna* extracts can be considered as a potential drug-like candidate in indigenous system of traditional medicine against trematode infections in livestock.

**Graphical abstract:**

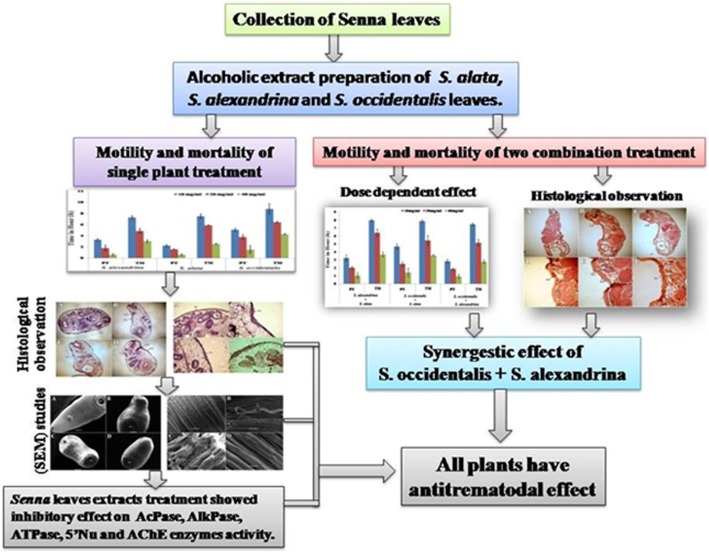

## Background

Paramphistomosis is a neglected parasitic disease of ruminants caused by one of the most common trematode parasites *Paramphistomum sp*. It has a worldwide geographical distribution with the highest prevalence in different regions of tropical and subtropical regions, particularly in Africa, Asia, Australia and Europe [1–4]. The disease paramphistomosis is characterized by acute parasitic gastroenteritis with very high morbidity and mortality rates, especially in livestock [5–10]. Slaughterhouse based survey study has shown that about 46.2% of sheep and 30.9% of goats were infected with *Paramphistomum sp*. [11]. It has been projected that more than 500 million cattle are at risk annually due to paramphistomosis infection, and about 80–90% of death occurred among young ruminants due to *Paramphistomum sp.* infection [12, 13]. Paramphistomosis, in domestic ruminants effects in the Indian subcontinental region next to fasciolosis [14]. Mortality reaches above 30% in cattle and nearly 75% in sheep and goats due to high rate of paramphistomosis [15–17]. In some regions of India, South Africa, and Australia, mortality reaches up to 80 to 90% in sheep and cattle [18, 19]. Nevertheless, recent reports demonstrated that *Paramphistomum daubneyi* in Europe can cause significant gastrointestinal disease, which drops in yield or even death in ruminants [20]. Most infections of adult fluke are harmless, although pathogenicity of paramphistomosis depends on the number of parasites in the animals, which cause a chronic ulcer in ruminal papillae [21]. Migration of adult worms in duodenal mucosa causes severe enteritis, possibly organ tissue destruction, and tissue hemorrhage that are responsible for anorexia, polydipsia, unthriftiness, muscle fibrosis, severe diarrhea, and ultimate mortality [1].

Acute paramphistomosis is caused by *Paramphistomum sp.* infection in the small intestine of young (less than 2 years of age) and immune deficiency goats, which effects on the organs, is characterized by listlessness, strangulation, haemorrhage, and anorexia [22–24]. Rastogi et al. [25] showed that goats aged below 1 year had a higher prevalence rate (18.5%) than those between 1 and 3 years (15.9%) and above 3 years (15.6%). The propensity of goats to higher infection rates in young age groups can be attributed to higher susceptibility and lower resistance due to incomplete development of immunity [25]. Higher infection rates in female goats than the males could be attributed to genetic predisposition and differential susceptibility owing to hormonal effects [26].

Paramphistomosis has also recently emerged as a major cause of productivity loss in livestock [27]. The disease is characterized by acute parasitic gastroenteritis with high morbidity and mortality rates, lower nutrition conversion, decreased milk production and reduction of fertility resulting in greater economic loss in the dairy industry and meat production in developing countries, like India [28]. Subclinical paramphistomosis infection is not properly diagnosed, due to lack of proper noticeable symptoms with which it is usually associated [29]. Presently, very limited trematodal drugs or molecules are available for the treatment against paramphistomum infection. Bithionol sulfoxide was reported being highly effective, although the resistance of rumen flukes to this drug was also noted [30]. Others available drugs have inherent disadvantages, such as side-effects, narrow spectrum, high cost, poor availability. In addition, resistance developed in most of these drugs due to parasitic adaptation facilitated by irrational use. To avoid harmful side effects of these synthetic antitrematodal drugs, it is essential for us to promote the studies of traditionally used anthelmintic plants which can lead to the development of new sustainable, effective and safe alternative antitrematocidal drugs.

Medicinal plants used by ethnic groups worldwide have been shown to possess anthelmintic activity using modern scientific tools. As per the World Health Organization, 80% of the world’s population relies on traditional medicines for their primary health care. Medicinal plants have been used as valuable drugs, either as crude extracts or in the form of molecules. The use of medicinal plants in developing countries has grown considerably in the second half of twentieth century [31] and often viewed as a strategy by these nations to reduce import of drugs, thereby boosting economic self-reliance [32]. *Senna* comprises 580 species of herbs, shrubs, and trees, which are widely distributed throughout the world, of which only 20 species are indigenous to India [33]. Many of *Senna* species has been reported of medicinal properties e.g. - *S. alata* has been recognized for centuries in traditional medicine for its antimicrobial [34], antifungal [35], purgative [36], anti-inflammatory [37], analgesic [38], antitumor [39], and hypoglycaemic activities [35]. *S. alexandrina* is known for its purgative properties and for regularization of bowel movements [40] while *S. occidentalis* has been traditionally used for the treatment of constipation, skin diseases, and as laxative and diuretic [41]. All three species belonging to the family Fabaceae and have been validated for their anthelmintic properties including in cestodes for the first time [42–47]. However, despite usage in similar conditions, to the best of our knowledge, these species have not been investigated for their activity against trematodes.

Though anthelmintic studies have been reported in cestodes (*Hymenolepis diminuta*, *Raillietina tetragona)* and nematodes (*Heterakis gallinarum*) [44–46], but in trematode is being left untouched. The tegument is an important part of trematodes that is in direct contact with tissue and body fluids of the host’s, that act as an interface between the host and parasite to carries out a number of functions which maintain the ionic homeostasis, absorption of nutrients, osmoregulation, protection against the host’s digestive enzymes and immune responses. Understanding the structural organization of the tegument is essential in developing any rational drugs or vaccines, which may damage the parasites through their actions on the tegument. Fasciolosis is well documented for its worldwide distribution and the serious economic losses caused by trematode parasites *Fasciola hepatica* in the animal husbandry industry. Despite few observations on the fine surface features of *Paramphistomum* parasites, is closely associated with *Fasciola hepatica* [48–50]. Base on the present fact, our aim of this study was to (1) evaluate the potential antitrematodal activity of three species of *Senna* plants (*Senna alata, Senna alexandrina* and *Senna occidentalis*) ethanolic leaves extracts on *Paramphistomum gracile* in terms of paralysis and death of parasites, (2) Investigation of alteration or damage in tegument architecture of *Paramphistomum gracile* by histomorphological and scanning electron microscopy (SEM) due to action of *Senna* treatments, (3) The effects of three *Senna* plants extracts on tegument enzymatic activity of *Paramphistomum gracile*.

## Results

### In vitro motility assessment

**Fig. 1:**
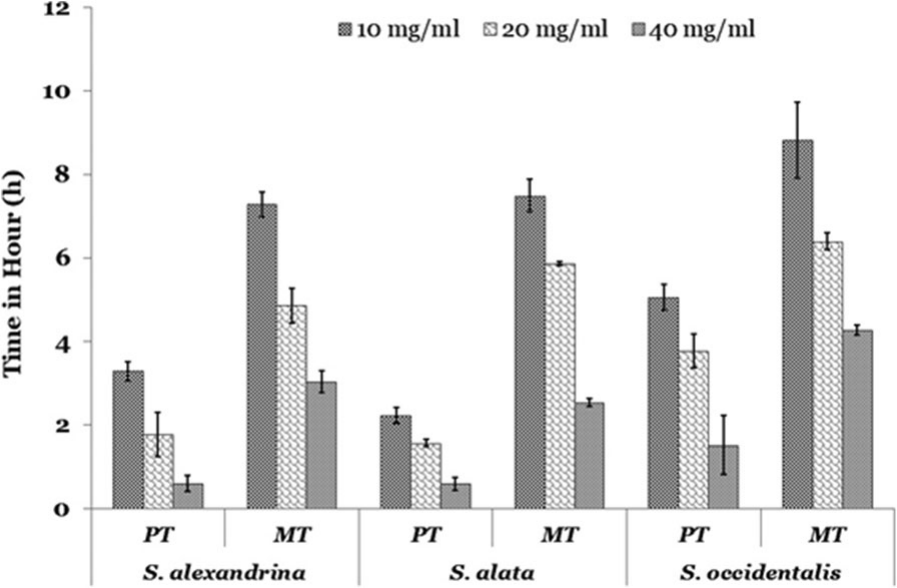
In vitro effect of three different *Senna* plants ethanolic leaf extracts on the motility and mortality of *Paramphistomum gracile.* Values are expressed as mean ± SE for each group (*n* = 5). Survivability of parasites in control medium 43.53 ± 2.29 h. PT- time of paralysis; TM- time of mortality

**Fig. 2:**
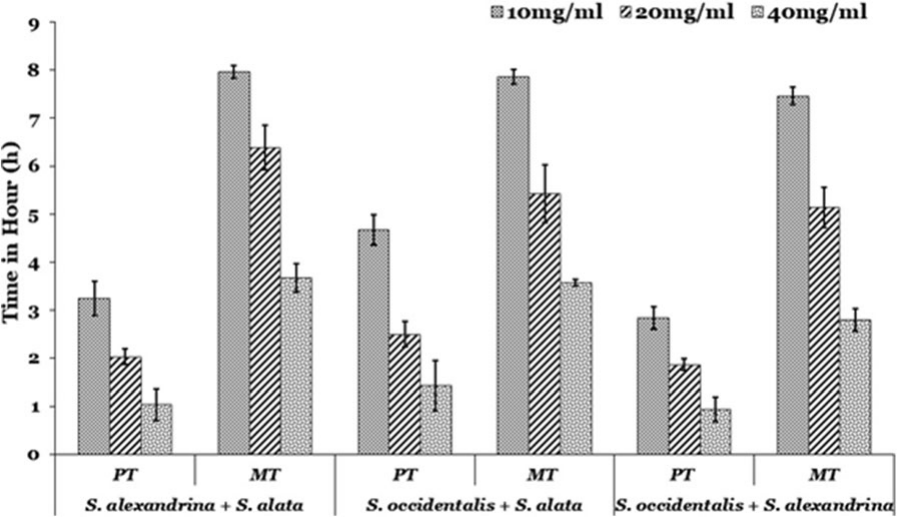
In vitro effect of three different Senna plants ethanolic leaf extracts in combination (1:1) of two plants on the motility and mortality of *Paramphistomum gracile*. Values are expressed as mean ± SE for each group (*n* = 5). Survivability of parasites in control medium 43.53 ± 2.29 h. PT- time of paralysis; TM- time of mortality

The results indicate that the parasites were paralyzed and finally death occurred after incubation in different concentrations of three *Senna* extracts with high significant value (*p* < 0.001) (Fig. 1). The present study showed maximum efficacy observed in *Senna alata* extract compared to *Senna occidentalis* and *Senna alexandrina*. Reduction of the time duration for paralysis and death time was observed with an enhancement of the doses in a dose-dependent fashion. At highest concentration (40 mg/mL) of treatment, paralysis (PT) occurred after 0.6 ± 0.15 h, 0.61 ± 0.2 h and 1.52 ± 0.7 h in *S. alata, S. alexandrina,* and *S. occidentalis* incubation respectively. Similarly, mortality (TM) or death of parasite occurred at 2.54 ± 0.1 h, 3.03 ± 0.26 h, and 4.28 ± 0.12 h, after treated with *S. alata*, *S. alexandrina,* and *S. occidentalis* respectively at 40 mg/ml concentration. While the control parasites stay alive in control medium till 43.53 ± 2.29 h. The post-paralytic time in all the three *Senna* extracts tested was comparatively shorter. In the three combinations treatment worms get paralysed at 1.03 ± 0.33 h, 1.0 ± 0.25 h, and 1.42 ± 0.52 h after treatment with (*S. alexandrina* + *S. alata*), (*S. occidentalis* + *S. alexandrina*) and (*S. occidentalis* + *S. alata*) respectively in 40 mg/mL concentration. The same pattern was revealed in mortality (Fig. 2). In comparison with the single extract treated parasites, the combination treated parasites required more time for paralysis as well as in death. Thus the highest efficacy was observed in individual plant treatment.

### Histological observation

**Fig. 3:**
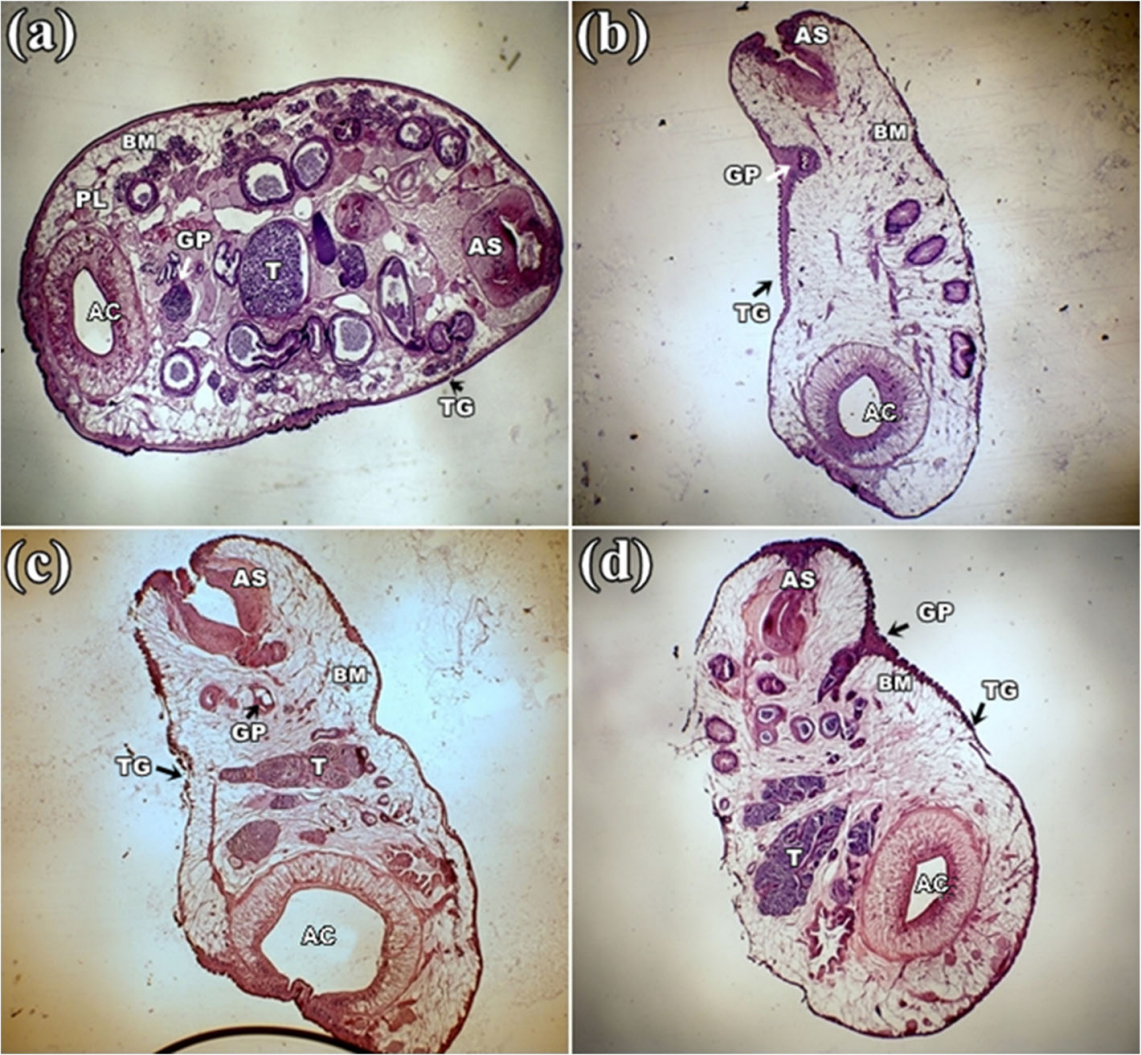
Histological micrograph of *Paramphistomum gracile* tegument. **a** Control parasite: Smooth tegument (TG) with alternating grooves and folds, anterior sucker (AS), acetabulum (AC) adjacent to ovary (O) and testis (T) and a gonopore (GP); **b** *Senna alata*, **c** *Senna alexandrina* and **d** *Senna occidentalis* treatment: Body tegument with deepened grooves and increase in folds. Shrinkage in anterior sucker (AS) and acetabulum (AC). All magnification at 4X

**Fig. 4:**
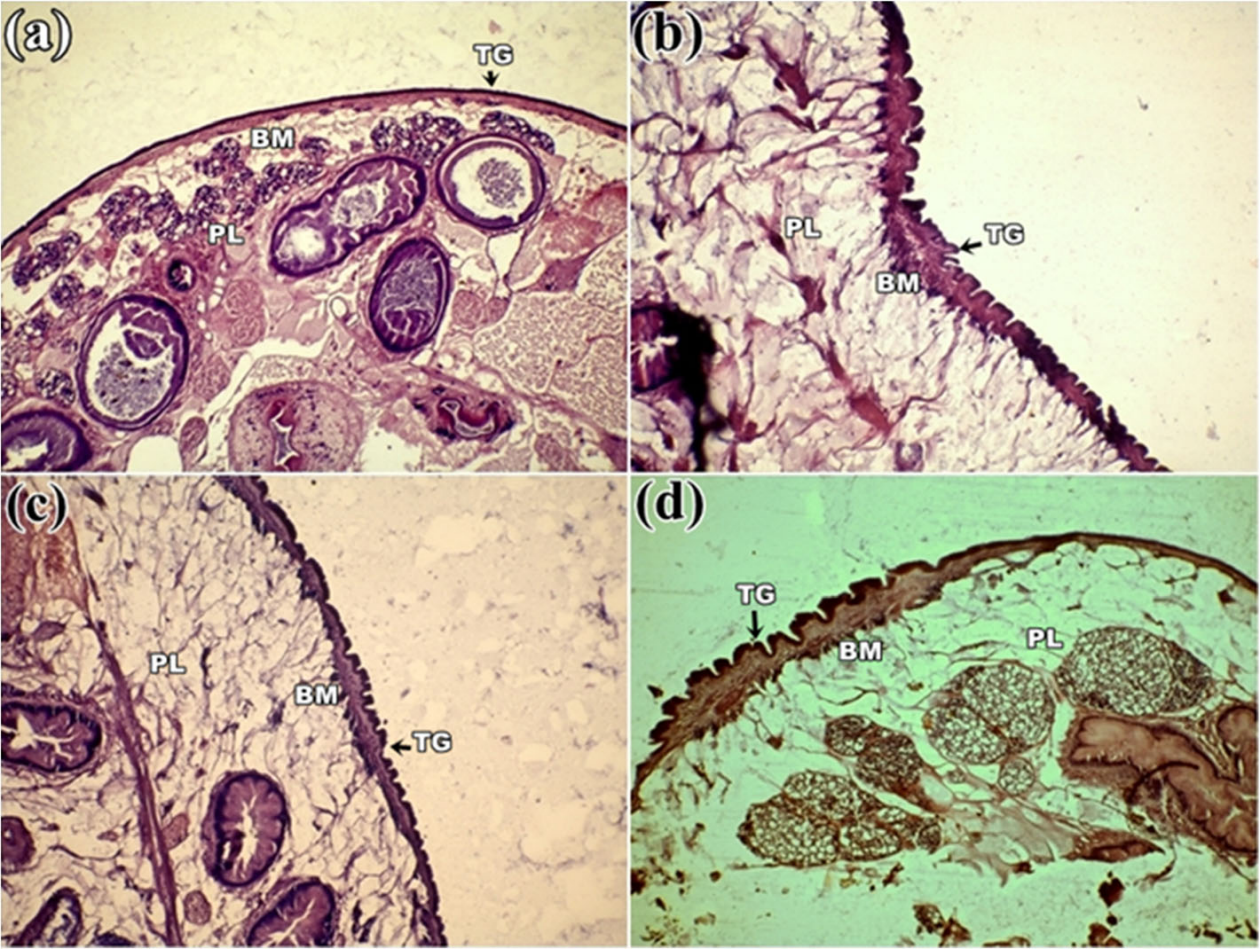
Histological micrograph of the tegument *Paramphistomum gracile* **a** Control parasite: Smooth tegument (TG) followed by a uniform basal membrane (BM) and dense parenchymal layer (PL) with few vitelline cells (VC); **b** *Senna alata*, **c** *Senna alexandrina* and **d** *Senna occidentalis* treatment: showing multiple grooves and folding’s in the outer tegument, with irregular folding’s in the basal membrane and lucent parenchymal layer. All magnification at 10X

**Fig. 5:**
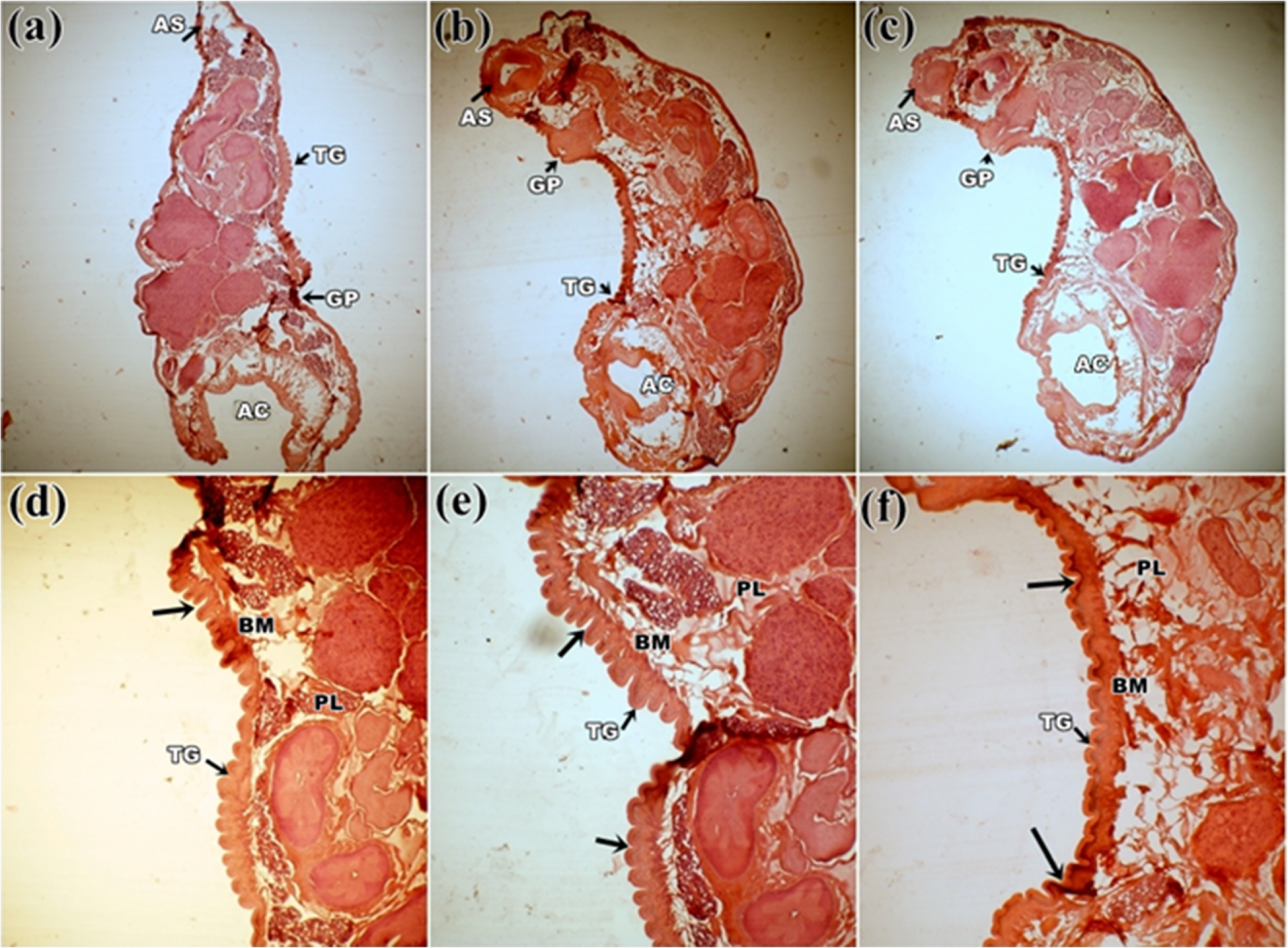
Histological micrograph of *Paramphistomum gracile* treated with a combination of two *Senna* plants ethanolic leaf extracts. **a** *Senna alexandrina* + *Senna alata*, **b** *Senna occidentalis + Senna alata*, **c** *Senna occidentalis + Senna alexandrina*: Showing immense shrinkage of the parasite and constriction in both the oral and acetabulum (AC). All magnification at 4X.: **d** *Senna alexandrina* + *Senna alata*, **e** *Senna occidentalis + Senna alata*, **f** *Senna occidentalis + Senna alexandrina*: At higher magnification showing deep and multiple folding’s of the outer tegument (TG), folding’s in the basal membrane and lucent parenchymal layer. All magnification at 10X

Control parasite revealed the normal structure of the body wall with folds and alternating grooves as a continuous homogeneous surface layer covering the entire body. Distinct anterior and posterior sucker located at two opposite ends. A pair of testis (T) and an ovary (O) was seen situated close to the posterior sucker (Fig. 3a). Prominent vitelaria was observed in both lateral sides of the body. In contrast, all the parasites treated with plants extracts were found reduced in size through the length and the homogenous body wall shrinkage accompanied by tegumental disruption. Damaged and disrupted internal organs were found in this groups with the maximum damage noticed in the group treated with *S. alata* (Fig. 3b) followed by *S. occidentalis* and *S. alexandrina* (Fig. 3c, d). Most of the organs totally get damaged, and the anterior and posterior sucker was constricted and loss the actual shape. At high magnification, the basement membrane (BM) of the tegument in the control parasite showed clear uniform layer, below which occupied distinct parenchymal layer (PL) and vitelline cells (VC) (Fig. 4a). After treatment with all three plant extracts, the uniformity of outer homogenous surface layer of the parasites was totally disrupted, resulted into numerous folds, deep grooves, furrows, and the parenchymal layer became lucent compared to control. However, tegumental as well as internal cellular damage was observed more in *S. alata* treated parasite (Fig. 4b) followed by *S. occidentalis* and *S. alexandrina* (Fig. 4c, d). In combination, therapy of *Senna* plant extracts, similar morphological damages were observed in the body surface tegument and internal organ. In each combination treatments, shrinkage in the whole body as well as in acetabulum and the anterior/oral sucker was observed clearly (Fig. 5a, b, and c). At higher magnification, it was observed that the tegument revealed many folds and deep grooves throughout the body surface. In *S. alexandrina + S. alata* and *S. occidentalis + S. alata* combination treatment basement membrane (BM) get thine and the parenchyma layered (PL) as well as vitelline cells (VC) loss their integrity while treated with *S. occidentalis + S. alexandrina* combination treatment parenchyma layered (PL) as well as vitelline cells (VC) were found totally distorted (Fig. 5d, e, and f).

### Scanning electron microscope study

**Fig. 6:**
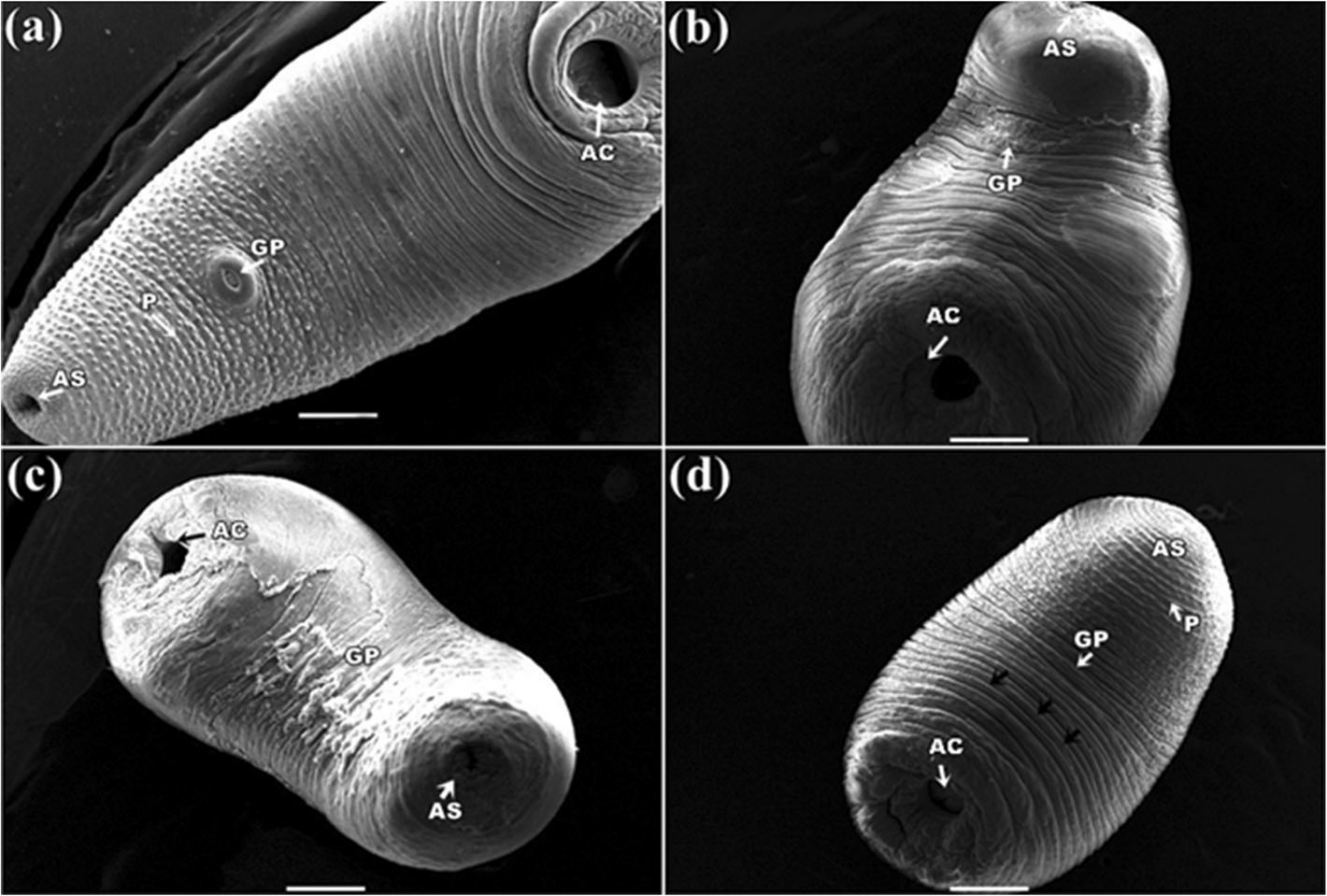
Scanning electron micrograph of *Paramphistomum gracile:* **a** Control parasite: showing small anterior sucker (AS), gonopore (GP) positioning one third of the body, and large acetabulum (AC), numerous papillae (P) at the anterior region; **b** *Senna alata* treatment showing shrinkage in the whole parasite with ballooning (→) on the body surface; **c** *Senna alexandrina*: showing shrinkage parasite with tearing (→) of the body surface and **d** *Senna occidentalis*: Shrinkage of the parasite with no noticeable change in the body surface

**Fig. 7:**
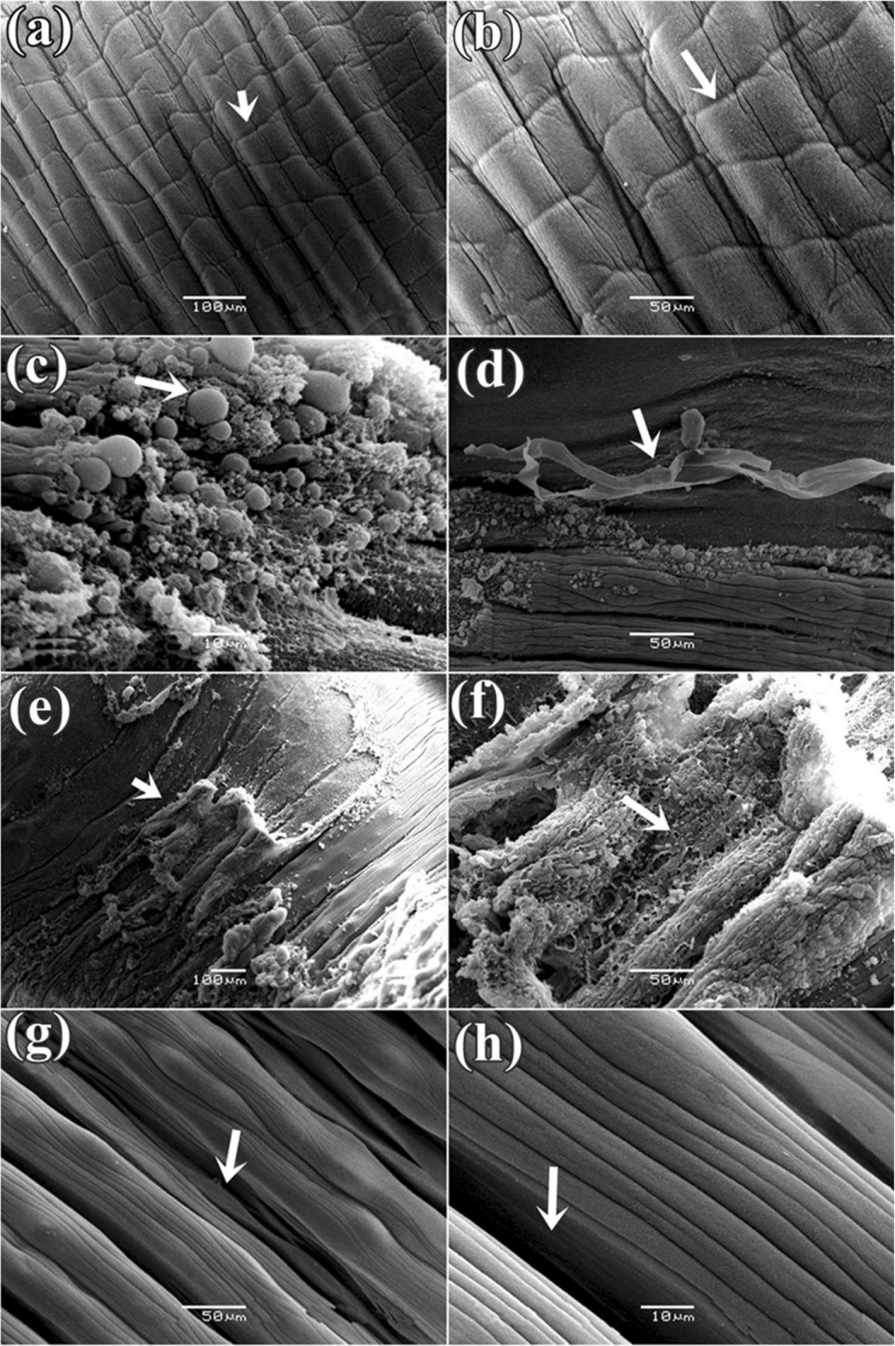
Scanning electron micrograph of tegument of *Paramphistomum gracile:* **a** Control parasite: showing trapezoid (→) shaped tegument; **b** at higher magnification no tegumental disruption observed and **c** & **d** *Senna alata* treatment showed tegumental blebbing and peeling off the outer tegumental layer **e** & **f** *Senna alexandrina*: showing tearing (→) of the tegument; **g** & **h** *Senna occidentalis*: showing few cracks (→) and loss of trapezoid architecture

**Fig. 8:**
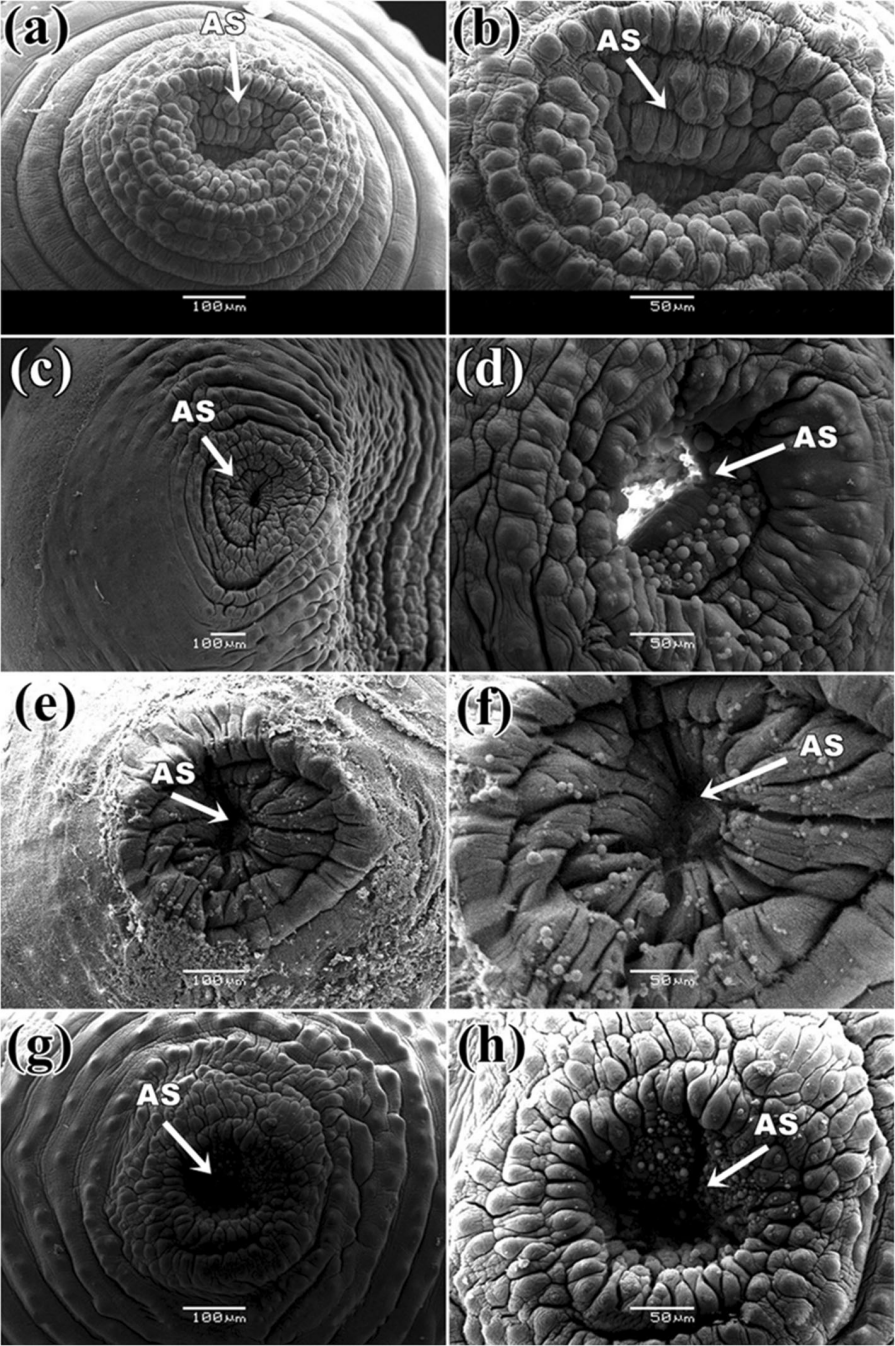
Scanning electron micrograph of anterior sucker of *Paramphistomum gracile*: **a** Control parasite: Showing wide sucker with defined circular rims (→) of the tegument and in **b** prominent distinct numerous papillae surrounded around sucker; **c** and **e** *Senna alata* and *Senna alexandrina*: showing constriction of sucker, in (**d** & **f**) higher magnification showed loss of regular rims and papillae were displaced and distorted; **g** & **h** *Senna occidentalis*: showing slight constriction of sucker, papillae get altered in structure while the circular rims displaced

**Fig. 9:**
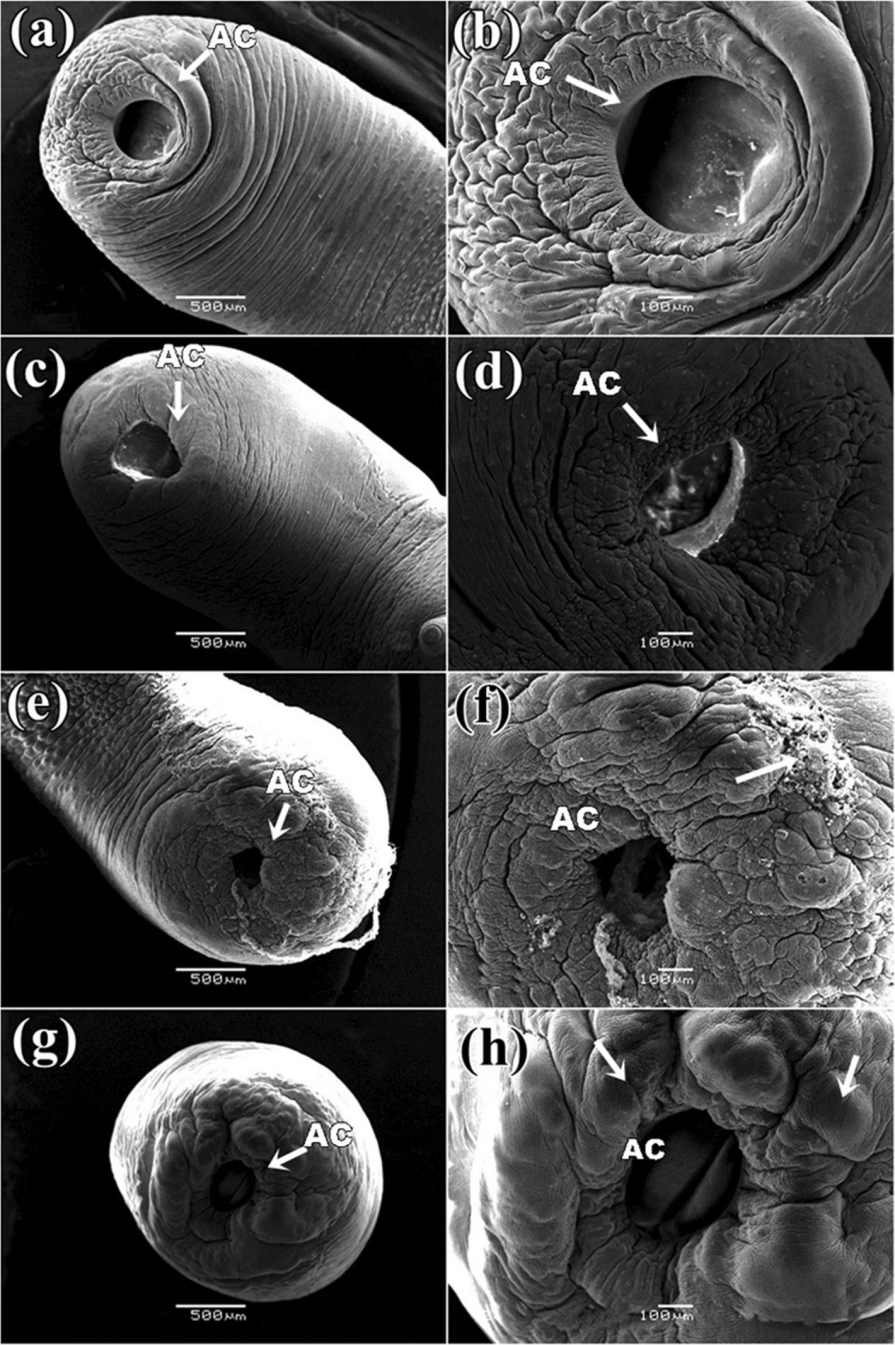
Scanning electron micrograph of posterior sucker of *Paramphistomum gracile*: **a** Control parasite: Showing wide sucker with thick tegument (→) **b** at higher magnification it showed thick muscular rim like structure around it; **c** & **d** and **e** & **f** *Senna alata* and *Senna alexandrina*: showing constriction of sucker and shrinkage of the tegument around it and proper architecture get distorted; **g** & **h** *Senna occidentalis*: showing constriction of sucker with ballooning (→) of the tegument around it

**Fig. 10:**
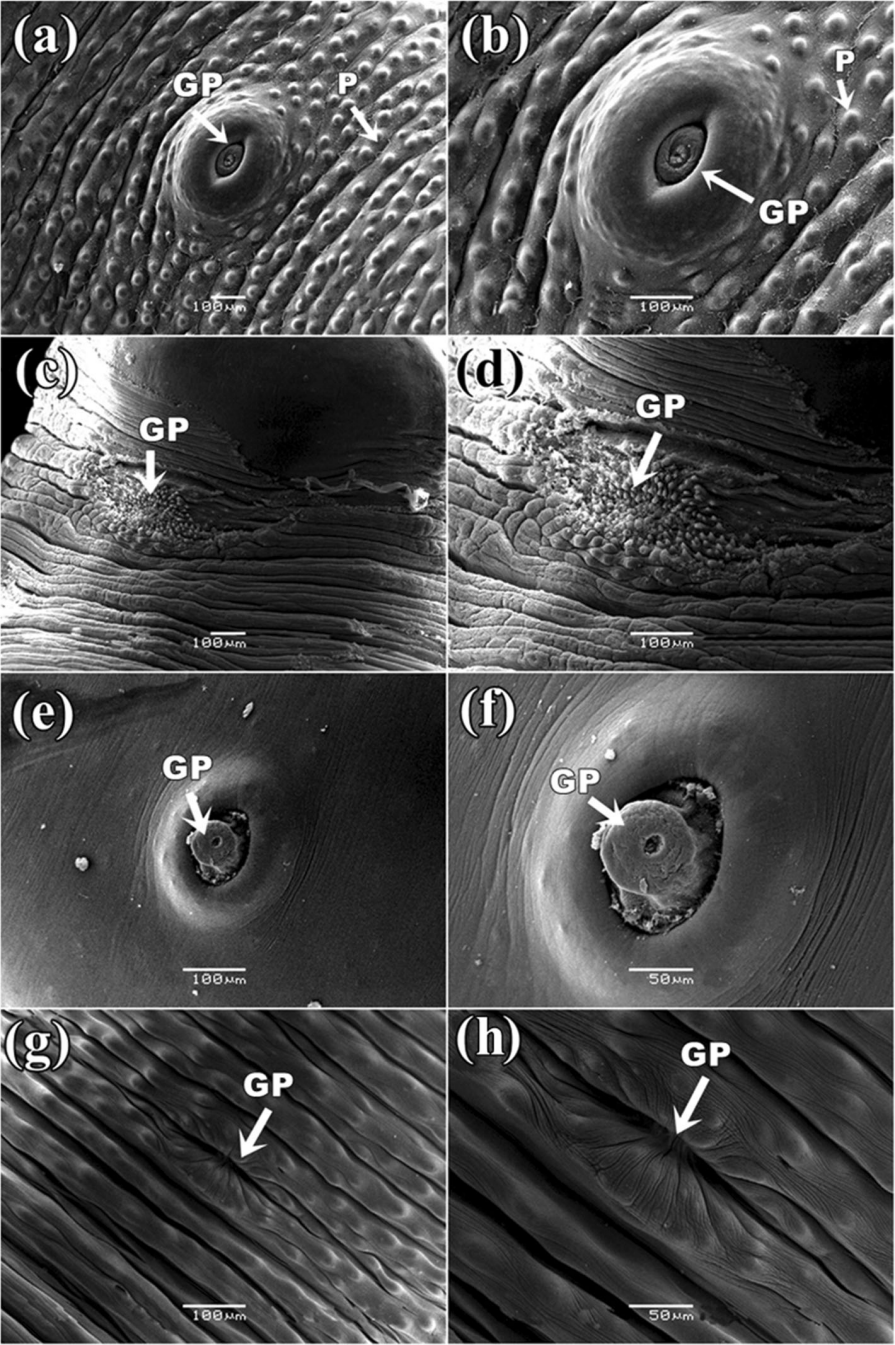
Scanning electron micrograph of genital pore of *Paramphistomum gracile*: **a** & **b** Control parasite: Showing thick tegument (→) around the pore and numerous papillae; **c** & **d**: *Senna alata* showing constricted pore and the tegument around it is torn and loss of papillae; **e** & **f** *Senna alexandrina*: showing evagination of pore and only a few unnoticeable papillae around it; **g** & **h** *Senna occidentalis*: showing constricted pore and papillae with irregular displacement of papillae

It was found that the control parasite has a prominent architecture with a pear-shaped body and a narrow anterior end having a small oral sucker. Presence of a large, more or less circular posterior sucker or acetabulum (AC), bound by a muscular rim around the opening along with scattered papillae. Small gonopore was seen at a position, one third from the anterior end of the body. The tegument contained numerous elevated papillae arranged in transverse rows and intensity is more at the anterior region of the body surface (Fig. 6a). After exposure in three *Senna* extracts, parasites body size reduced and shrinkage occurred through body length. It was observed that the texture of tegument was disrupted in *S. alata* treated parasite and highly shrinkage throughout the body (Fig. 6b). Similarly, tearing and shearing the body surface was noticed in *S. alexandrina* (Fig. 6c). However, in *S. occidentalis,* shrinkage and deep furrows were arises in the body surface tegument of the parasite (Fig. 6d). At higher magnifying observation revealed of several blabbing like out-growths in the surface tegument and shredding of the tegument from the body that of the parasites treated with, *S. alata* (Fig. 7c, d). In *S. alexandrina* (Fig. 7e and f) parasite showed irreparable destruction of the tegument as well spongy appearance was noted on the parasite body surface different from the control group (Fig. 7a, b). Minor damage was observed in parasite treated with *S. occidentalis* extract, it showed the presence of cracks and deep furrows in the tegument (Fig. 7g, h). SEM studies of control parasite revealed distinct oral sucker located at the anterior end of the body, which is transversely elongated with strong muscular boundary surrounded by well-organized numerous papillae (Fig. 8a, b). But in *S. alata* treated parasite anterior sucker lost the original architecture and reduced in proper size while the papillae were found dislocated and often disappeared (Fig. 8c, d). In case of *S. alexandrina* treated parasite, the mouth shape was modified and papillae were distorted (Fig. 8e, f). However in *S. occidentalis* extract treated parasite, surface tegument around the mouth was disrupted (Fig. 8g, h). The posterior sucker or acetabulum (AC) of the control worm consisted of a large round muscular rim, guarded with thick tegument with scattered papillae (Fig. 9a, b). After treatment the shape of the acetabulum (AC) gets disoriented and the tegument around it become shrinked after treated with *S. alata* (Fig. 9c and d), while swelling of the tegument occurs after treated with *S. alexandrina* (Fig. 9e, f) and *S. occidentals* extracts (Fig. 9g, h). Numerous distinct sensory papillae were found to surround the genital pore region in control parasite (Fig. 10a, b). However, the treated parasite genital pore reduced to a slit and no noticeable papillae were observed in the genital pore region of *S. alata* and *S. occidentalis* treated parasite (Fig. 10c, d, e, and f) while the genital pore evaginates in *S. alexandrina* treatment (Fig. 10g, h).

### Observation of plant extracts effects on tegumental enzymes

Biochemical assays revealed a significant decline in the activity of Acid phosphatase (AcPase), Alkaline phosphatase (AlkPase), ATPase and 5′-Nucleotidase (5′Nu) when compared to control. The particular effects of *Senna* extracts on tegumental enzymatic activity were given detailed in Table 1. From the obtained results, it was observed that in the control parasites AcPase unit activity 20.28 ± 2.68 unit/g wet wt. tissue/h and specific activity 0.65 ± 0.08 unit/mg protein. There was a pronounced decline in the AcPase enzyme activity observed for all treated parasites after exposure of *P. gracile* to the different *Senna* extracts. Among the three *Senna* extracts treatment, the highest reduction of AcPase activity was observed in *S. alata* treated parasites, where the AcPase unit activity was 10.35 ± 0.69 unit/g wet wt. tissue and specific activity was 0.30 ± 0.01 unit/mg protein. It has calculated about 53.9% reduction occurred in AcPase activity in comparison with control. Similarly in *S. occidentalis* and *S. alexandrina* treated parasites AcPase total unit activity was 12.51 ± 2.94 and 14.23 ± 3.35 unit/g wet wt. tissue and specific activities were 0.36 ± 0.08 and 0.43 ± 0.09 unit/mg protein respectively. So, it has calculated that 44.6% inhibition occurred in specific activity in *S. occidentalis* treatment and 33.9% in *S. alexandrina* treated parasite with comparable with control (Table 1).

**Table 1:** Effects of three different *Senna* plants leaf extracts on *Paramphistomum gracile* in the unit and specific activity of tegumental enzymes (AcPase, AlkPase, ATPase, 5′-Nu, and AChE)

	Control	*Senna alata*	*Senna occidentalis*	*Senna alexandrina*
AcPase				
Unit activity	20.28 ± 2.68^a^	10.35 ± 0.69^b^	12.51 ± 2.94^b^	14.23 ± 3.35^b^
Specific activity	0.65 ± 0.08^a^	0.30 ± 0.01^d^	0.36 ± 0.08^c^	0.43 ± 0.09^b^
Inhibition in %	0	53.9	44.62	33.85
AlkPase				
Unit activity	343.97 ± 5.67^a^	175.55 ± 2.12^d^	187.78 ± 2.98^c^	203.63 ± 6.9^b^
Specific activity	1.09 ± 0.02^a^	0.54 ± 0.01^c^	0.55 ± 0.02^c^	0.62 ± 0.01^b^
Inhibition in %	0	50.5	49.54	43.12
ATPase				
Unit activity	936.22 ± 20.15^a^	627.82 ± 27.52^d^	782.24 ± 23.97^c^	869.76 ± 31.52^b^
Specific activity	2.98 ± 0.06^a^	1.94 ± 0.05^d^	2.23 ± 0.06^c^	2.55 ± 0.18^b^
Iniabition in %	0	34.9	25.17	14.43
5′- Nu				
Unit activity	1581 ± 25.66^a^	1159.5 ± 101.79^a^	1525.05 ± 33.49^b^	1573.95 ± 4.5^a^
Specific activity	5.03 ± 0.08^a^	3.54 ± 0.31^c^	4.17 ± 0.01^b^	4.76 ± 0.01^a^
Inhibition in %	0	29.6	17.10	5.37
AChE				
Unit activity	473.82 ± 21.55^a^	397.06 ± 51.45^a^	412.06 ± 55.33^a^	433.23 ± 54.69^a^
Specific activity	11.66 ± 1.11^a^	9.41 ± 1.22^b^	9.57 ± 1.3^b^	11.39 ± 1.42^a^
Inhibition in %	0	19.3	18.02	2.32

Values are given as mean ± SE from five replicates assays (*n* = 5). Unit enzyme activity expressed as a specific unit which consumes 1.0 M substrate/g wet weight tissue/h. Specific activity = unit activity/mg tissue protein /h. Different small letters ^(a, b, c, d)^ on the error value indicate significant at (*P* < 0.001) among the treated worm as well as with the control. Same letters indicate no significant differences between the same variable between different plant extracts treatment

Furthermore, for AlkPase total unit activity in the control parasites was observed as 343.97 ± 5.67 unit/g wet wt. tissue and specific activity as 1.09 ± 0.02 unit/mg protein. Enzymatic activity decreased significantly after treatment with the three different *Senna* extracts. From the obtained result it was observed that the highest decline in AlkPase activity was clearly remarkable in *S. alata* treated parasites followed by *S. occidentalis* and *S. alexandrina.* It was found that *S. alata* treated parasite AlkPase unit activity 175.55 ± 2.12 unit/g wet wt. tissue and specific activity 0.54 ± 0.01 unit/mg protein. Thereby, it was showing about 50.5% inhibition occurred due to *S. alata* treatment. Similarly, significant decreases were also noticeable in *S. occidentalis* and *S. alexandrina*, where unit activity was 187.78 ± 2.98 and 203.63 ± 6.9 unit/g wet wt. tissue, as well as specific activity was 0.55 ± 0.02 and 0.62 ± 0.01 unit/mg protein respectively. So, it was calculated that about 49.54 and 43.12% depletion occurred in AlkPase specific activity in parasites after treatment with *S. occidentalis* and *S. alexandrina* respectively, comparable with control (Table 1).

Similarly, significant depletion occurred in ATPase enzyme activity after treatment with *Senna* extracts comparable with control. In control parasite, ATPase unit activity was 936 ± 20.15 unit/g wet wt. tissue and specific activity was 2.98 ± 0.06 unit/mg protein. While after treatment with *S. alata*, *S. occidentalis,* and *S. alexandrina* extracts significant depletion in ATPase enzymatic activity were remarkable in treated parasite body. From obtained results, it was found that *S. alata*, *S. occidentalis,* and *S. alexandrina* extracts treated parasite ATPase unit activities were 627.82 ± 27.52, 782.24 ± 23.97 and 869.76 ± 31.52 unit/g wet wt. tissue respectively and specific activities were 1.94 ± 0.05, 2.23 ± 0.06, and 2.55 ± 0.18 unit/mg protein respectively. ATPase specific activity was significantly decreased by 34.9 and 25.17% in *S. alata* and *S. occidentalis*, and comparatively lesser decreased by 14.43% in *S. alexandrina* comparable with control (Table 1).

Furthermore, depletion in 5′Nu enzymatic activity also observed in *Senna* extracts treated parasites, compared with control. In control parasite, 5′Nu unit activity was 1581 ± 25.66 unit/g wet wt. tissue and specific activity was 5.03 ± 0.08 unit/mg protein. After *S. alata* treatment 5′Nu unit activity was observed 1159.5 ± 101.79 unit/g wet wt. tissue and specific activity was 3.54 ± 0.31unit/mg protein, so 30% inhibition occurred. However, in *S. occidentalis* treated parasite only 17% inhibition occurred in 5′Nu activity, while *S. alexandrina* did not show significant enzyme inhibition in unit activity (1573.95 ± 4.5 unit/g wet wt. tissue) as well as specific activity (4.76 ± 0.01 unit/mg protein) comparable with control. From the obtained result it was observed that highest decline in 5′Nu activity was clearly remarkable in *S. alata* treated parasites. In the case of AChE activity, control parasite AChE unit activity was observed 473.82 ± 21.55 unit/g wet wt. tissue and specific activity were 11.66 ± 1.11 unit/mg proteins. The AChE activity in the parasite tissue decreases significantly about 20% after treatments with *S. alata* and *S. occidentalis* extracts, while only a little decrease in activity (of about 3%, not significant) was observed in treatment with *S. alexandrina*. Here also *S. alata* showed highest inhibitory effects among the three plants (Table 1).

## Discussion

The present investigation illustrates that the three species of *Senna* extracts have potential antitrematocidal action against *Paramphistomum gracile*. Concentration-dependent effects of *Senna* extracts on parasitic motility and mortality were found individually as well as in combination treatment. Similar type of observations were also reported by Bashtar et al., and Ferreira et al. [51, 52] that individual and combination treatment of different plant extracts (*Artemisia cina, A. annua, A. absinthium, Asimina triloba*, and *Fumaria officinalis*) showed dose-dependent trematocidal activity on *Moniezia spp*, *Thysaniezia spp*, *Schistosoma mansoni*, *Fasciola hepatica*, and *Echinostoma caproni* parasites in vitro. Amongst the three species of *Senna* plants, *S. alata* caused earliest paralysis followed by *S. alexandrina* and *S. occidentalis* respectively. However, *S. occidentalis* treatment has taken longer time to paralyse the worms by itself, but in combination with *S. alata* and with *S. alexandrina* it reduced the time of paralysis (PT) and time of mortality (TM) significantly (*P* < 0.001). This may be suggested that in combination the two plants may possess a synergistic effect and the combined action may have an efficacious intervention on the target organisms, like that observation also noticed by Kundu et al., in *Hymenolepis diminuta* treated with different *Senna* plants [43]. These variations in efficacy may be responsible for the presence of different active chemical constituents or secondary metabolites at variance concentration into the different *Senna* extracts [53]. Similar observations were recorded in our earlier studies when *Hymenolepis diminuta* was treated with *Senna* leaf extracts [43, 45]. Though no early mortality was depicted in lowest concentration (10 mg/mL) for all *Senna* extracts treatment, still there was a considerable significant paralysis occurred. It may therefore, be suggested that all *Senna* extracts singly or in combination possibly exert a reversible action on the neuromuscular system of the parasite, and though it did not cause early mortality, but once paralyzed, it took very short time for mortality to commence. Thus, it may be suggested that it possesses a vermifugal activity in nature and the inactiveness caused would last long enough for the parasites to be swept out of host’s body [42–45]. Therefore, from this study, we may assess that, *Senna* extracts have the potential effects of *P. gracile* in motility and mortality.

For evaluating anthelmintic action, histopathology and scanning electron microscopy has proved the first and most obvious pathological effects of plant treatment on the parasite. The tegument is an important structure of trematode parasite because it provides covering and protection of the parasite’s body, and supports internal organs. It also controls the secretion, synthesis, perception of sensory stimuli and osmoregulation. Therefore, histomorphology and electron microscopic observations of *Senna* extracts treated parasites revealed that remarkable changes occurred on tegumental surface, with extensive shrinkage and consisted of swelling, blebbing, which was later ruptured, leading to erosion, perforation, and desquamation of the tegument, resulting in the lesion, and finally the exposure and disruption of basal lamina. A similar type of disruptions noticeable in *Paramphistomum cervi* after treatment with Plumbagin is a compound of Plumbago indica/rosea [54]. Depletion of the parenchymal tissue observed in treated worms may be contributed to the decrease in total glycogen content [55]. Pronounced vacuolization and surface blebbings in the tegument of the *Senna* extracts treated parasites may be attributed on the ionic homeostasis and neural impulse conductance of worm muscle membrane producing hyperpolarization and reducing excitability that could lead to muscle relaxation and flaccid paralysis, which caused would lead the treated parasite to be swept out of the host’s tissue [43, 45, 51, 54]. This can be regarded as a stress response resulting from emergency repair to a damaged tegument that may be induced by many harmful events [56]. Once the body tegument is totally destroyed, the drug-like candidate could penetrate deeper into the muscular layer and caused motility reduction and cessation that lead finally to death. Similar incidents happen in *P. gracile* after treatment with the three *Senna* extracts treatment. Disorientation and loss of density of sensory papillae are indications of disruption in sensory system of the parasite [57]. And disorientation in the gonopore in all treated worms may result in failure in reproduction [58–60]. The papillae were also damaged by *Senna* leaves extracts treatment which could cause the loss of sensory functions. Besides, the changes were found at the oral sucker and acetabulum, which exhibited the swollen appearance and scattered blebs along their rims. Such pronounced destruction affects the attachment ability with the host tissues [61, 62].

Tegumental enzymes (AcPase, AlkPase, ATPase and 5′-Nu) have an important role in maintaining ionic homeostasis, active transport and also regulation in metabolic processes within the parasite [63]. Alkaline phosphatase and adenosine triphosphatase take part in active transport through cellular membranes and acid phosphatase deals with intracellular digestion processes. So it can accept potential drug target or therapeutic strategy to combat parasitic infections. Therefore, alteration in enzymatic activity of AcPase, AlkPase, and ATPase in *P. gracile* by three *Senna* plants may affect in absorption, intracellular digestion of food items, and membrane transportation in the parasite tissue. Similar finding also observed in tegumental enzymes activities in *Raillietina echinobothrida* after treatment with *Acacia oxyphylla, Securinega virosa* and synthetic drug praziquental [64]. Furthermore, ATPases enzyme activity is known to be related to energy metabolism, by catalyzing the synthesis of energy in form of ATP, which plays an important role in active ion transport across plasmalemmal membranes, neuromuscular propagations, etc. [65]. Thus, 50% inhibitions of ATPase activity occurred in *P. gracile* due to *Senna* extracts treatment, it denotes the less production of ATP and neuromuscular disability in the parasite, which promotes early paralysis and death of parasite. The decrease of the enzyme’s activity may be occurred due to disruption of the absorptive surface. Similar observations have been reported in a trematode after treatment with mebendazole and levamisole [66]. In helminthes, 5’nucleotidase enzyme plays a key role in the metabolism of nucleotides and hydrolysis of pyrimidine and purine bases [65, 67]. Therefore 30% reduction in 5′-Nu activity on *P. gracile* affects the metabolism of nucleotides in the parasite body, which affects egg production in the parasite [67]. The neurotransmitter enzyme, AchE is an important enzyme which is found to be primarily associated with neuromuscular system and neuromuscular motor activity of the parasites [68]. Therefore, the present study reveals that inhibition of the AchE activity promotes relaxation in the musculature of the parasite by inhibiting rhythmical movements and eventually producing paralysis in treated parasite, leading to loss of attachment ability in the host intestine and hence a vermifugal action may ensure. A similar type of inhibitory effect has also been reported in other helminths [69].

## Conclusion

In conclusion, from our present study, we discover that ethanolic leaf extracts of *S. alata, S. alexandrina,* and *S. occidentalis* can be used as an antitrematodal drug, which possesses strong antitrematodal activity against ruminant parasite *P. gracile* based on in vitro screening assay. Furthermore, histomorphological and ultrastructural observation showed that *Senna* extracts inflict structural alteration on the tegument and also showed inhibitory effects on tegumental enzymes activity. Taken in conjunction with our present study, it was announced that three *Senna* plants have strong potentiality as a drug like candidate for the treatment of paramphistomum infection.

## Methods

### Collection and identification of the plants

Fresh leaves of *Senna alata* (L.) Roxb., *Senna occidentalis* (L.) Link., and *Senna. alexandrina* Mill. grown widely surrounding the Visva-Bharati University campus, were collected during the winter season. The herbariums were prepared for identification of these three plants species and voucher specimens have been deposited in the Central National Herbarium, Botanical Survey of India, Kolkata, India, with voucher No. VBSL-1, VBSL-2, and VBSL-3 for *Senna alata* (L.) Roxb, *Senna occidentalis* (L.) Link, and *Senna alexandrina* Mill. respectively.

### Preparation of ethanolic leaf extracts

Fresh leaves of three plants species of *Senna* were collected separately. The leaves were washed thoroughly with deionized water, allow it to air-dry. For total drying, the leaves were kept under hot-air oven at 45°C temperature for 1 hour (h). Dried leaves were crushed into powder and kept in airtight containers as labeled separately for further use for extract preparation. For the ethanolic extract preparation, about 250 g of every type powder sample was added in 1 L of 90% ethanol in Soxhlet apparatus (Borosil soxhlet apparatus, Model No. 3840029) for 8 h. The decoction was filtered through Whatman filter paper. The filtrate was then concentrated as a semisolid mass under vacuum at 40°C using a rotary evaporator (EYELA Rotary evaporator, Model: N-1110 V-W). The extract (yield =10% w/w of the initial powder) was stored at 4°C until use for evaluation of antitrematocidal activity.

### Parasite collections and identification

Live parasites *(Paramphistomum gracile)* were collected from different goats rumen from various local slaughterhouses in the Santiniketan campus (Westbengal, India) throughout the year around and were washed thoroughly with 0.9% phosphate-buffered saline (PBS) at pH 7.4. The parasites were deposited in the Zoological Survey of India with Accession No. W0772/1.

### Experimental design

In vitro anthelmintic assessment was performed according to the Kundu et al. [42] with slight modifications. In brief, Five parasites (*n* = 5) were incubated with different concentration (10, 20 and 40 mg/mL) of alcoholic leaf extracts of *S. alata*, *S. occidentalis,* and *S. alexandrina* dissolves in 10 mL of PBS (pH -7.4) with 1% dimethylsulfoxide (DMSO) in a covered glass Petri plates respectively. For combination treatment, assessment was done according to the Kundu et al. [43], where parasites (*n* = 5) were incubated with different concentrated (10, 20, 40 mg/mL dose) of the combined extracts of *Senna* (*S. alata* + *S. occidentalis*, *S. alata* + *S. alexandrina* and *S. occidentalis* + *S. alexandrina)* in (1:1) ratio dissolved in 10 ml of PBS with 1% dimethylsulfoxide (DMSO) in a covered glass Petri plates separately. One group of parasites (*n* = 5) were maintained only in 10 mL of PBS with 100 μL of 1% DMSO as a control group. The experiments were carried out at 37°C temperature with 60% humidity in a humidity incubator. Motility and mortality time was recorded during this experiment. Time for paralysis (PT) was observed at different time intervals when no movement of worms could be observed except when shaken vigorously or kept in warmer PBS (40°C temperature). Time of mortality (TM) or death was recorded at the time when worms showed no movement, even when shaken vigorously or put into slightly hot PBS.

### Histological studies

Immediately after paralysis, treated parasites from the various treatments groups (single and in combination treatment) were fixed in Bouin’s fixative as a prospective type of treatments for 24 h. Subsequently processed for histological study after proper fixation following the method of Drury & Wallington [70]. In brief, the parasites were washed and dehydrated by graded ethanol series (30–100%) at room temperature and embedded in paraffin. Tissues were then cut into thin sections from tissue embedded paraffin block by using microtome. Then sections were stained with Haematoxylin and Eosin before observation under a light microscope.

### Scanning electron microscope (SEM) studies

Immediately after paralysis, all the treated and control parasites were fixed for scanning electron microscope (SEM) studies following standard method Roy and Tandon [71]. In brief, all type of treated parasites and control parasites were fixed in 3% glutaraldehyde prepared in 0.1 M cacodylate buffer (pH 7.4) at 4°C for 2 h. Then parasites were dehydrated with ascending grades of acetone and then air-dried in tetramethylsilane (TMS) and finally observed under the Jeol JSM 6360 scanning electron microscope at an electron accelerating voltage of 20 kV.

### Estimation of tegumental enzymes assay

For assaying the activity of tegumental enzymes, immediately after paralysis single leaf extract at 40 mg/ml dose treated parasites and control parasites were collected and kept into − 20°C for further biochemical estimations.

### Estimation of acid phosphatase (AcPase) and alkaline phosphatase (AlkPase) activity

Acid phosphatase (AcPase) and Alkaline phosphatase (AlkPase) activity were assayed by estimating the p-nitrophenol product following the method abducted from Plummer [72] with slight modification as described by Pal and Tandon [73]. In brief, a 10% (weight/volume) tissue homogenate was prepared in 125 mM sodium acetate and glacial acetic acid (pH 4.5) and centrifuged at 5000 rpm (revolutions per minute) for 20 min. The supernatant was used for enzyme assay. While for AcPase, 1 mL of assay mixture contained 125 μM sodium acetate buffer (pH 4.5), 62.5 μM P-nitrophenyl phosphate and enzyme source 0.2 ml. For the AlkPase activity estimation, the reaction mixture contained 0.1 μM of sodium glycine buffer (pH 10.5), 31.25 μM of p-nitrophenyl phosphate and 0.1 mL of enzyme source. The mixture was incubated at 37°C for 20–30 min. After incubation, the reaction was stopped by adding 5 mL of 0.02 N NaOH. Absorbance measured at 405 nm.

### Estimation of adenosine triphosphatase (ATPase) activity

Adenosine triphosphatase (ATPase) activity was assayed by estimating the free phosphate released following the method of Kaplan [74] with slight modification as described by Pal and Tandon [73]. 10% (w/v) of parasite tissue homogenate was prepared in glycine buffer (0.2 M, pH 9.1) with Triton-X and sonicated for 30 s. The final assay mixture contained 200 μM glycine buffer (pH 9.1), CaCl_2_ (100 μM), Na-ATP (100 μM) and enzyme source (0.05 mL). One milliliter of 15% trichloroacetic acid (TCA) was added into the reaction mixture, continuation with centrifugation at 3000–4000 rpm for 10 min. After completion of the centrifuge, 0.25 mL of parasite tissue supernatant was added with a mixture of 0.75 ml distilled water, 0.5 ml 5 N H_2_SO_4_, 0.5 mL ammonium molybdate (2.5% w/v) and 0.1 ml freshly prepared Fiske and Subbarao reducer. The reaction mixture was incubated for 10 min at 37°C and the absorbance was taken at 700 nm.

### Estimation of 5′ Nu activity

The 5′ Nu activity was assayed by estimating the free phosphate released following the method described by Bunitian [75] with slight modification as described by Pal and Tandon [73]. Tissue homogenate (10%, w/v) was prepared in Tris-HCL buffer (pH 7.45). The assay mixture contained 40 μM Tris-HCL buffer (pH 7.45), MgSO_4_ (12 μM), AMP (5 μM) and 0.1 ml of 10% tissue homogenate as enzyme source. After that 1.5 ml of 10% (w/v) (PCA) was added to the reaction mixture for 1 h. Precipitated protein was separated out by centrifugation at 3000 rpm for 10 min at room temperature and the supernatant was collected. Then 0.5 ml water, 0.5 ml 5 N H_2_SO_4_, 0.5 mL ammonium molybdate (2.5% w/v) and 0.1 mL freshly prepared Fiske and Subbarao reducer were added to the supernatant. The reaction mixture was incubated for 10 min at 37°C temperature and absorbance was taken at 700 nm.

### Estimation of acetylcholine esterase (AChE) activity

Acetylcholine esterase (AChE) activity was estimated following the method of Ellman et al. [76] with slight modification as described by Pal and Tandon [73]. A 10% tissue homogenate was prepared in 0.2 M sucrose solution and centrifuged at 20,000 g at 4°C for 30 min and the supernatant was used as an enzyme source. The assay mixture in a final volume of 3 mL contained of 150 mM sodium phosphate buffer (pH 7.4), acetylthiocholine iodide (10 mM), 5–5′ dithiobis nitrobenzoic acid (DTNB, 1.25 mM), triton X-100 (0.3%) and enzyme extract (0.1 mL). After proper mixing of all ingredients, the assay mixture was incubated at 37°C for 5 min. The absorbance of the assay mixture measured at 412 nm and the rate of AChE enzyme activity calculated by the specific activity of enzyme expressed in terms of nmoles thiocholine produced/mg protein in the reaction mixture. Thiocholine concentration of the sample was calculated using an extinction coefficient of 13,600/M/cm.

### Protein estimation and specific activity

Protein was estimated following the method of Lowry et al. [77], using bovine serum albumin as standard. The specific activity of the enzymes was expressed as the units of enzyme activity per mg protein (unit activity/mg protein). A 10 μL protein sample pipetted out to different test tubes and distilled water was added making up a volume to 1 ml. About 2.5 mL of Lowry reagent was added and gently mixed. This solution was incubated at room temperature for 10 mins. Further, 250 μL of diluted Folin Ciocalteau reagent (at 3:1 ratio) solution was added to each tube and incubated for 30 min. Then colorimeter was set at Zero as blank and the absorbance was measured at 660 nm. The absorbance against protein concentration was plotted to get a standard calibration curve. The absorbance of the unknown sample was noted and its concentration was determined using the plotted standard curve.

$$ \mathrm{Specific}\ \mathrm{activity}=\frac{\mathrm{Unit}\ \mathrm{activity}\ \mathrm{of}\ \mathrm{particular}\ \mathrm{enzyme}}{\mathrm{Concentration}\ \mathrm{of}\ \mathrm{protein}\;\left(\frac{\mathrm{mg}}{\mathrm{ml}}\right)\;\mathrm{of}\ \mathrm{that}\ \mathrm{particular}\ \mathrm{sample}} $$

### Statistical analysis

Data are expressed as mean values ± standard error (SE) for each group (*n* = 5). For determining statistical significance, standard error and analysis of variance (ANOVA) at 95% level of significance were employed considering *P* < 0.001 as significant. Evaluation of statistical significance between the control and experimental data set was carried out by Duncan’s Multiple Range Test (DMRT). Different small letters on the error bars indicate significant (*P* < 0.001) differences in the values of a particular variable between the different treated group and control. Same letters indicate no significant differences within these groups.

## Data Availability

The datasets used and analysed during the current study are available from the corresponding author on reasonable request.

## Abbreviations

AMP: Adenosine monophosphate
AMP: Adenosine monophosphate
h: Hour
H_2_SO_4_: Sulfuric acid
MgSO_4_: Magnesium sulphate
Na-ATP: Adenosine triphosphate in Sodium salt
NaOH: Sodium hydroxide
nm: Nanometre
PCA: Perchloric acid
w/v: Weight/volume
wt: Weight
μM: Micromole
